# Nurse-Led Large Language Model Chatbot for Predicting and Preventing Complications After Coronary Artery Bypass Grafting: Protocol for a Randomized Controlled Trial

**DOI:** 10.2196/103717

**Published:** 2026-08-04

**Authors:** Salini K, Ajee K L, Praveen Kerala Varma, Premjith B, Anila K P, Vandana Agnihotri, Priyalatha Muthu, Renjitha Bhaskaran, Namrata Nair

**Affiliations:** 1 Department of Medical Surgical Nursing, Amrita College of Nursing, Amrita Vishwa Vidyapeetham, Amrita Institute of Medical Sciences and Research Center, Kochi Campus, Ponekkara, Edappally, Kochi, Kerala, 682041, India, 91 8335053920; 2 Department of Cardiovascular and Thoracic Surgery (CVTS), Amrita School of Medicine, Amrita Vishwa Vidyapeetham, Kochi, Kerala, India; 3 Department of Artificial Intelligence, Amrita School of Artificial Intelligence, Amrita Vishwa Vidyapeetham, Coimbatore, Tamil Nadu, India; 4 Department of Medical Surgical Nursing, College of Nursing, Armed Forces Medical College, Pune, Maharashtra, India; 5 Department of Medical Surgical Nursing, College of Nursing, RAK Medical & Health Sciences University, Al Juwais, Ras Al Khaimah, United Arab Emirates; 6 Amrita Institute of Medical Sciences, Amrita Vishwa Vidyapeetham, Kochi, Kerala, India; 7 School of Computing, Amrita Vishwa Vidyapeetham, Amrita Technologies, Amritapuri, Kollam, Kerala, India

**Keywords:** coronary artery bypass grafting, large language model, hospital readmission, artificial intelligence, remote patient monitoring, electrocardiographic monitoring, randomized controlled trial, postoperative care, postoperative complications, thirty-day hospital readmission

## Abstract

**Background:**

Thirty-day unplanned readmission following coronary artery bypass grafting (CABG) affects 10%-20% of patients and is a key quality indicator, particularly in low- and middle-income countries (LMICs) where access to cardiac rehabilitation is limited. Existing risk models are static, lack real-time engagement, and no validated large language model (LLM)–based clinical decision support (CDS) system exists for post-CABG readmission prevention.

**Objective:**

This protocol describes the development, validation, and evaluation of Smart CABGuard, a nurse-led, LLM-based CDS chatbot with continuous remote electrocardiographic (ECG) monitoring, to predict and prevent postoperative complications, and 30-day unplanned readmission after isolated CABG.

**Methods:**

This multiphase translational study is conducted at Amrita Institute of Medical Sciences, Kochi, India, following TRIPOD-LLM (Transparent Reporting of a Multivariable Prediction Model for Individual Prognosis or Diagnosis–Large Language Model) and CONSORT (Consolidated Standards of Reporting Trials) AI reporting guidelines. Phase I uses an ambispective design (retrospective: January 2020 to December 2024; prospective needs survey: June 2025 to January 2026) to develop a logistic regression-based Complication Risk Index (CRI), assessed via receiver operating characteristic area under the curve, Brier score, and decision curve analysis. Phase II refines Smart CABGuard by integrating a frozen Mistral-7B LLM (low-rank adaptation fine-tuned), the CRI engine, explainable AI, and single-lead ECG telemetry (Amrita Spandanam device), with a usability threshold of ≥80%. Phase III is a prospective, parallel-group, open-label randomized controlled trial (RCT). Adults aged ≥18 years undergoing isolated CABG with smartphone access are randomized 1:1 via permuted block randomization, with allocation concealment. The intervention arm receives Smart CABGuard–assisted care (daily chatbot check-ins, CRI-based risk stratification, ECG monitoring, and nurse-led triage) for approximately 24 days postdischarge plus standard care; the control arm receives standard care with structured telephone follow-up for outcome ascertainment only. Outcome assessors and statisticians are blinded; analyses follow the intention-to-treat principle. The primary outcome is 30-day all-cause unplanned readmission. Based on a baseline rate of 10.71%, a 50% relative reduction, α=.10, and 80% power, the target sample size is 800 patients (400 per arm, including 10% attrition).

**Results:**

Ethics approval was granted by the Institutional Ethics Committee of Amrita Institute of Medical Sciences on April 16, 2025. Funding was awarded by Sigma Theta Tau International Honor Society of Nursing, Small Grants Program (grant 21650) in June 2025. Phase I data extraction commenced in May 2025 and is projected to be completed by April 2026; the patient needs survey (June 2025 to January 2026) is ongoing. Phase II usability evaluation is projected from May to July 2026. Phase III recruitment is anticipated from August 2026 to September 2027. Results are expected to be published in early 2028.

**Conclusions:**

Smart CABGuard is the first RCT protocol of an LLM-based nurse-led CDS system for post-CABG readmission prevention in an LMIC setting, aiming to establish the usefulness, safety, and feasibility of AI-augmented postoperative surveillance.

## Introduction

Cardiovascular diseases are the leading cause of death worldwide, accounting for about 32% of all global deaths and placing a heavy burden on health care systems in terms of cost and loss of productive life [1,2]. Among patients with severe coronary artery disease, coronary artery bypass grafting (CABG) is the most effective treatment, and it has been shown to significantly improve survival, physical function, and quality of life [3-5]. The relevance of CABG extends well beyond high-income countries. In India, rapid urbanization, an aging population, physical inactivity, and rising rates of diabetes and hypertension have sharply increased the burden of coronary artery disease over recent decades [6-8]. As a result, large tertiary cardiac centers in India now perform thousands of CABG surgeries each year.

While deaths during and immediately after CABG surgery have decreased significantly due to improvements in surgical technique, anesthesia, and intensive care, patients continue to face serious risks after hospital discharge. The early postdischarge period is particularly vulnerable [9]. Patients transition from a closely monitored hospital setting to a home environment, where they must manage their own recovery—including wound care, medication management, blood sugar control, and gradual physical activity—simultaneously [10]. During this period, life-threatening complications such as atrial fibrillation, heart failure, pleural effusion, wound infections, kidney dysfunction, and fluid and electrolyte imbalances can develop quietly, often without obvious warning signs until the patient deteriorates significantly [11].

Mental health problems also play an important role. Anxiety, depression, poor sleep, and low adherence to medications are common after CABG and can further worsen recovery outcomes [12]. Many of these early complications and setbacks are potentially preventable if patients and nurses are supported with timely information, symptom monitoring, and early intervention. This gap between hospital discharge and the first follow-up visit is where the greatest opportunity for intervention lies.

Unplanned readmission within 30 days of CABG surgery is recognized internationally as an important marker of care quality, patient safety, and continuity of care [13-15]. Reported 30-day readmission rates after CABG range from 10% to 20%, with considerable variation between hospitals and health systems [14,16-18]. The most common reasons for readmission include surgical site infections, arrhythmias, fluid overload, respiratory complications, kidney impairment, and poor medication adherence.

A large proportion of these readmissions are considered preventable with structured discharge planning, early symptom identification, proactive nurse-led follow-up, and timely clinical action. Readmissions also create significant emotional and financial strain for patients and families. In low- and middle-income countries (LMICs) such as India, where out-of-pocket medical costs are high and social support systems are limited, even a single readmission can cause severe financial hardship [19,20]. Reducing preventable readmissions, therefore, directly improves both patient well-being and health care efficiency.

Standard cardiac rehabilitation and structured follow-up programs are known to improve outcomes after CABG, but participation in LMICs is low due to geographic distances, limited access to specialists, financial constraints, and a shortage of trained staff. This makes the need for technology-based, scalable solutions that can work beyond hospital walls even more pressing.

Over the past decade, several risk prediction models have been developed to identify patients at high risk for specific post-CABG complications, including atrial fibrillation, surgical site infections, prolonged mechanical ventilation, acute kidney injury, and readmission and costs. These models use methods such as logistic regression, random forests, and neural networks. While some have shown reasonable performance in their development datasets, their adoption in routine clinical practice remains limited for several important reasons [21-26].

Most of these models rely on data collected at a single point in time—usually at or before surgery—and cannot account for how a patient’s condition changes day by day after discharge. Many have not been validated in populations outside the centers where they were developed, making them unreliable in other settings, especially LMICs. Their predictions are also difficult for clinicians to interpret because the models do not explain which factors are driving the risk score, making clinicians less likely to act on them [25]. Crucially, none of these models can accept patient-reported symptoms in free-text form, provide personalized guidance, or support ongoing nurse-patient interaction during the recovery period.

Large language models (LLMs) represent a major step forward in the use of AI in health care. Unlike conventional machine learning tools that work only with structured, numerical data, LLMs can process diverse types of information—including clinical notes, laboratory results, and patient-written descriptions of symptoms—and generate contextually appropriate clinical responses [27-30]. This makes them uniquely suited for the postdischarge period, where symptoms are often described in everyday language and do not fit neatly into structured forms.

When LLMs are built into conversational chat interfaces, they allow for continuous interaction between patients and the system, enabling real-time symptom monitoring, personalized responses, and dynamic risk assessment. There is growing evidence supporting LLM-based systems in clinical settings, including decision support in oncology, nurse-led treatment planning in Indian district hospitals, and clinical education using virtual patient simulations. A recent rapid review also highlighted the growing use of generative AI tools in nursing practice and clinical care delivery [31-34]. Despite these developments, no LLM-based clinical decision support (CDS) system has yet been developed or validated to predict and prevent 30-day readmissions and complications specifically after CABG surgery—and certainly not for use in LMIC settings where such tools are most needed [35].

An effective post-CABG decision support system would need to bring together structured surgical data, ongoing clinical information from electronic health records (EHRs), and patient-reported symptoms to give clinicians and nurses accurate, real-time, and easy-to-understand risk assessments. It should also support discharge planning, promote patient education, and maintain continuity of care after discharge. A nurse-led LLM chatbot platform is particularly well suited for this role because it combines risk prediction, patient communication, and clinical guidance within a single accessible tool that does not require specialist availability.

This protocol describes the development and evaluation of a nurse-led, LLM-based CDS chatbot designed to predict and prevent 30-day complications and readmissions after CABG surgery. The system combines a multivariable risk prediction model with an LLM-driven conversational interface and continuous electrocardiographic (ECG) monitoring to provide nurses and clinicians with real-time, explainable, and actionable clinical information. The study incorporates stepwise model development and validation, explainability analysis, and a prospective randomized controlled trial (RCT), alongside structured evaluation of usability, nurse and clinician acceptance, patient satisfaction, and feasibility of implementation in a real-world cardiac care setting. This work directly addresses the limitations of existing static risk tools—particularly their lack of continuous monitoring, patient interaction, and clinical interpretability—and seeks to establish a practical, scalable approach for deploying LLM-based decision support in post-CABG nursing care.

Therefore, the primary objective of this study is to develop and evaluate the Smart CABGuard nurse-led AI chatbot to reduce 30-day unplanned readmissions after CABG surgery. Specifically, the study will (1) identify risk factors for post-CABG readmission; (2) compare the predictive performance of conventional multivariable logistic regression and LLM-based prediction for 30-day readmission; (3) assess patient usability, accessibility, and satisfaction with the chatbot; and (4) examine differences in postoperative morbidity patterns within 30 days between intervention and control groups.

## Methods

### Study Design and Reporting Framework

This investigation is designed as a multiphase, mixed methods translational study to develop, validate, and evaluate a nurse-led, LLM-based CDS chatbot (voicebot) integrated with continuous remote ECG monitoring for postoperative management following CABG. The study follows a structured 3-phase framework: (1) phase I: development of the risk prediction model and assessment of patient needs, (2) phase II: refinement of the system and evaluation of usability, and (3) phase III: prospective RCT.

**Figure 1.**
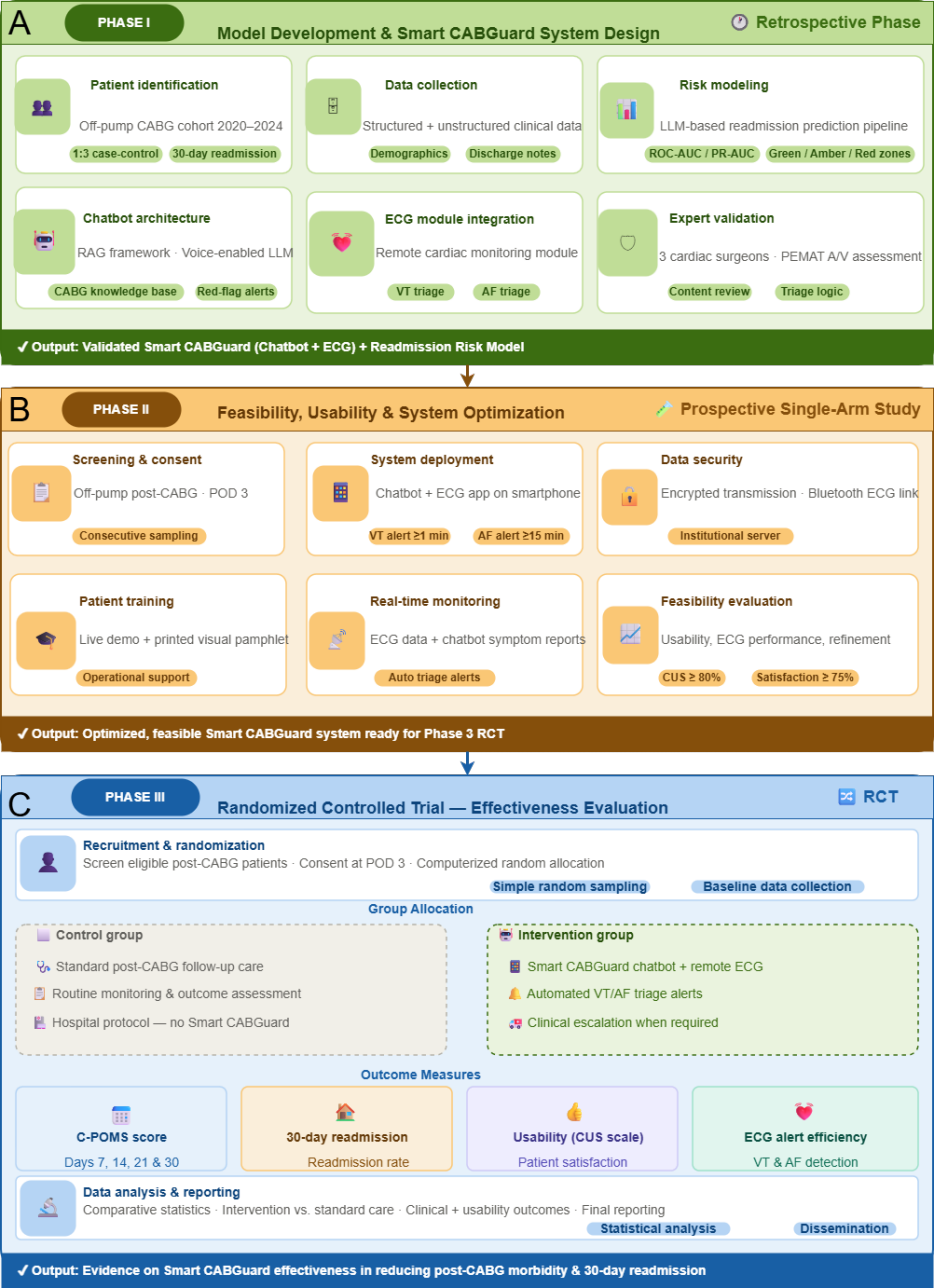
Overview of the smart cabguard multiphase study framework: (A) phase I (risk model development and system design), (B) phase II (feasibility, usability, and system optimization), and (C) phase III (randomized controlled trial for effectiveness evaluation). AF: atrial fibrillation; CABG: coronary artery bypass grafting; C-POMS: Cardiac Postoperative Morbidity Score; ECG: electrocardiographic; LLM: large language model; PEMAT A/V: Patient Education Materials Assessment Tool for Audiovisual Materials; POD 3: postoperative day; PR-AUC: precision-recall area under the curve; RAG: retrieval augmented generation; RCT: randomized controlled trial; ROC-AUC: receiver operating characteristic area under the curve; VT: ventricular tachycardia.

This 3-phase design allows for stepwise model development, clinical validation, and evaluation under real-world conditions. An overview of the 3-phase framework is presented in Figure 1. The trial phase is designed in accordance with CONSORT (Consolidated Standards of Reporting Trials) AI recommendations for randomized trials of digital health interventions, and the overall protocol adheres to internationally recognized reporting standards for AI-based CDS systems.

Phase-specific reporting guidelines will be followed throughout: phase I will adhere to the TRIPOD-LLM (Transparent Reporting of a multivariable prediction model for Individual Prognosis Or Diagnosis–Large Language Model) checklist, phase II to the mERA (mHealth Evidence Reporting and Assessment) checklist, and phase III to CONSORT 2010 and CONSORT AI extension. The completed checklist will be submitted as supplementary appendices.

### Trial Registration Details

This RCT is registered with the Clinical Trials Registry of India, approval number CTRI/2025/06/088163. The trial is sponsored by Amrita Institute of Medical Sciences, Kochi, Kerala, India. Any modifications related to AI architecture, chatbot functionality, ECG monitoring workflows, or data-handling procedures will undergo additional ethics review. This document constitutes Protocol version 1.0, dated June 2026. The primary endpoint for registration purposes is 30-day all-cause unplanned hospital readmission after isolated CABG; key secondary endpoints include time to detection of complications, postoperative morbidity, usability, patient satisfaction, and health service usage.

### Study Setting

The study will be conducted in the Department of Cardiovascular and Thoracic Surgery at a high-volume tertiary care hospital, Amrita Institute of Medical Sciences, Kochi, Kerala, performing more than 1500 CABG procedures annually. The institution comprises advanced cardiothoracic surgical infrastructure, dedicated cardiac intensive care services, structured discharge-planning programs, and an established digital health research ecosystem that enables secure data extraction, AI development, and clinical deployment.

### Multiphase Study Framework

#### Phase I—Risk Model Development and Patient Needs Assessment

##### Phase I Overview

Phase I adopts an ambispective design consisting of 2 distinct components. First, we will use a retrospective cohort from January 2020 to December 2024 to build and test the Complication Risk Index (CRI). Patients from 2020 to 2022 will be used to develop the model, and patients from 2023 to 2024 will be used to check its performance over time. Model performance, including discrimination, calibration, and clinical usefulness, will be evaluated and reported separately in the temporal validation subset before any prospective deployment of the CRI. The CRI coefficients and decision thresholds will be finalized only after completion of phase I temporal validation and a prospective calibration check during phase II. Once finalized, the CRI will be frozen and will not be modified during the RCT phase to ensure methodological consistency and reproducibility.

EHRs of adult patients who have undergone off-pump CABG will be reviewed, with the patients classified as cases (readmitted within 30 days) or controls (no readmission). A multivariable logistic regression model will be used, with predictors selected to achieve at least 10 events per variable to minimize the risk of overfitting.

##### Sample Size

For phase I prediction model development, we will use a retrospective cohort of approximately 1296 adults undergoing off-pump CABG between January 2020 and December 2024 at our institution, with an anticipated 75 unplanned 30-day readmissions (events) based on audit data. All available readmitted patients will be included as cases, together with about 225 nonreadmitted patients sampled from the remaining cohort to achieve an efficient 1:3 case:control ratio [36]. In line with contemporary guidance for clinical prediction models and TRIPOD recommendations, sample size adequacy is evaluated primarily on the number of outcome events rather than total sample size [37,38]. Classical events-per-variable rules suggest that at least 10 events per predictor variable are desirable to reduce overfitting in logistic regression; therefore, with ≈75 events, the final CRI will be restricted to a maximum of 7‐8 predictors, maintaining ≥10 events per variable [39,40].

The structured variables will include patient demographics, comorbidities, perioperative details, laboratory results, postoperative complications, discharge medications, and early clinical outcomes. Model performance will be assessed with measures of discrimination (receiver operating characteristic area under the curve [ROC-AUC] and precision-recall area under the curve [PR-AUC]), calibration, the Brier score, and decision curve analysis.

Second, an independent prospective cross-sectional survey (June 2025 to January 2026) will be conducted among post-CABG patients to assess informational needs, symptom burden, and self-care challenges. Findings from this component will inform the design of chatbot conversations, triage logic, and educational modules. These datasets will be analyzed separately to ensure that predictive model development remains methodologically distinct from qualitative and user-centered system design. Phase II will commence only after the CRI achieves acceptable discrimination (ROC-AUC ≥0.70) and calibration in the temporal validation cohort, and all decision thresholds are formally frozen. The key components of phase I are illustrated in Figure 1 (see phase I panel).

### Predeployment Clinician Evaluation of Chatbot Content

Before phase II usability testing, the chatbot’s core educational scripts and exemplar interactions will be reviewed by a panel of cardiothoracic surgeons and cardiac nurses using the Patient Education Materials Assessment Tool for Audiovisual Materials (PEMAT-A/V) [41], which rates understandability and actionability of patient education materials. Panel members will independently score a standardized set of chatbot conversations. In addition, to generate real-world interaction data before broader deployment, a minimum of 10 post-CABG patients will use the Smart CABGuard chatbot for 10 days following surgery, and the resulting interaction logs will be included in the PEMAT-A/V evaluation alongside the scripted exemplars. If either PEMAT-A/V domain score (understandability or actionability) falls below the predefined threshold of 80%, chatbot content will be iteratively revised until the target is achieved before patient-facing deployment in phase II.

### Phase II: System Refinement and Usability Evaluation

#### Phase II Overview

Phase II focuses on refining the Smart CABGuard platform and assessing its feasibility, usability, and acceptability. The intervention integrates a frozen, medically instruction-tuned LLM, a deterministic CRI engine derived from phase I, explainable AI modules, continuous remote ECG telemetry, and a nurse-led triage dashboard. Adult post-CABG patients will be enrolled before discharge and will undergo structured onboarding, including chatbot training and setup of a wearable ECG device, which enables continuous postoperative rhythm surveillance for early detection of arrhythmias such as atrial fibrillation and ventricular tachycardia—common causes of early post-CABG readmission [14], thereby supporting timely risk alerts and clinical escalation. Usability and satisfaction will be measured using the chatBot Usability Scale (BUS) [42] and the Client Satisfaction Questionnaire (8-item version) (CSQ-8) [43].

#### Sample Size

Phase II will enroll a minimum of 20 post-CABG patients for usability and satisfaction evaluation using the BUS and CSQ-8. This sample size follows established guidelines recommending at least 20 participants for quantitative usability studies to generate statistically meaningful scores [44] and is consistent with published chatbot usability and feasibility studies that typically include 15‐30 participants for pilot validation of a CBT-based mental health chatbot pilot RCT-enrolled 18 participants for feasibility and usability assessment [45], and health information chatbot evaluations commonly report samples of 20‐50 users [46]. Descriptive statistics and thematic analysis will inform refinements, with a usability threshold of ≥80% required before starting the RCT phase. The key components of phase II are illustrated in Figure 1 (see phase II panel).

### Phase III: RCT

#### Phase III Overview

Phase III (Figure 1, phase III panel) will be a prospective, parallel group, open-label randomized controlled trial that compares Smart CABGuard–assisted care with standard postoperative management. Eligible participants will be adults aged 18 years or older undergoing off-pump CABG, who can give informed consent and have access to a smartphone with internet. Patients who are having other major cardiac procedures (such as valve repair) at the same time, or who have severe cognitive impairment, a terminal illness, or who cannot complete follow-up, will be excluded. Participants will be randomized in a 1:1 ratio to Smart CABGuard plus standard care or standard care alone using a computer-generated permuted block sequence with variable block sizes prepared by an independent statistician. Sequentially numbered, opaque, and sealed envelopes will be assembled by a research assistant not involved in enrollment; after confirming eligibility and obtaining consent, the enrolling clinician will open the next envelope in sequence, thereby maintaining allocation concealment. Because of the nature of the intervention, participants and treating clinicians will not be blinded, but outcome adjudicators and statisticians will remain blinded to allocation. Randomization will not be stratified; any clinically important covariates that show baseline imbalance, defined as a standardized mean difference of >0.10 will be adjusted for in prespecified multivariable analyses. All randomized participants will be analyzed according to the intention-to-treat principle, and any deviations from the randomization procedure will be documented and reported. The intervention group will receive LLM-based chatbot-assisted postoperative monitoring, automated symptom surveillance, CRI-based risk stratification, continuous remote ECG monitoring, nurse-led triage and escalation, and structured digital education with daily chatbot check-ins, which will continue for approximately 24 days postdischarge, with automated alerts notifying nurses of high-risk symptom patterns or ECG-detected arrhythmias.

The research nurse will serve as the primary personnel providing the intervention, including triage and escalation based on chatbot alerts. In the absence of the research nurse (out-of-hours alert management), 2 trained research assistants will support the intervention under the researcher’s supervision to ensure continuity of care. The research assistants will undergo standardized training similar to that of the research nurse to maintain consistency in alert response, escalation thresholds, and documentation practices. There may also be variability in nurse triage and escalation despite standardized training and protocols, which could influence responsiveness to alerts; adherence to the triage algorithm will therefore be monitored as a process measure. The integrated post-CABG triage plan, including Green, Amber, and Red zone criteria for symptom domains, ECG alerts, wound care, and escalation pathways, is provided in Multimedia Appendix 1.

The control group will receive standard discharge instructions and routine outpatient follow-up according to institutional practice. In addition, brief structured telephone calls will be conducted on approximately postoperative days 7, 14, 21, and 30, primarily to document symptoms and capture readmission or emergency visits for outcome ascertainment and safety monitoring. These calls will follow a standardized script and will not provide individualized counseling, risk-stratified advice, or ECG-based monitoring, thereby avoiding replication of the intensity or decision support features of the Smart CABGuard intervention.

#### Sample Size

The estimated number of participants needed to achieve study objectives is based on the observed 30-day readmission rate of 10.71% following isolated CABG procedures at our institution. For the intervention arm, we assume a 50% relative reduction in readmission (intervention rate: 5.36%) supported by prior digital and telemonitoring interventions: the Perfect Care telemedicine program, which demonstrated a 75% relative reduction in 30-day readmissions after CABG (4% vs 16%) [47], and by remote patient monitoring after CABG showing a 44% reduction in 30-day readmission and mortality [48]. Smart CABGuard integrates continuous ECG monitoring, daily LLM-based symptom surveillance, and nurse-led triage, making a 50% reduction a conservative estimate relative to these comparators.

As this is an exploratory first RCT with no prior randomized evidence for LLM-based post-CABG monitoring, we use a priori power analysis with a 2-sided α=.10 for the primary outcome to reduce the risk of a false-negative result. With 80% power (1−*β*=.80), the required sample is 361 participants per arm. After allowing for 10% attrition, the final sample size is 400 participants per arm (total 800). Sample size was calculated using G*Power. A sensitivity analysis showing power under effect sizes of 35%, 50%, and 70% relative reduction is provided in Multimedia Appendix 2.

### Discontinuation of the Digital Intervention and Concomitant Care

Participants in the Smart CABGuard arm will receive all components of standard postoperative care in addition to the digital intervention. The chatbot and ECG monitoring may be paused or permanently discontinued for an individual participant in the following situations: (1) participant request or withdrawal of consent for the digital components; (2) persistent nonadherence, defined as no chatbot engagement for more than 7 consecutive days and/or failure to use or charge the ECG device despite at least 2 nurse-led reminders; (3) clinically relevant device-related problems such as significant skin irritation or intolerable discomfort; or (4) major technical failures that cannot be resolved within 72 hours and materially compromise monitoring. Discontinuation of Smart CABGuard will not limit access to usual clinical follow-up.

To reduce contamination, participants will be advised to avoid concurrent participation in other structured telemonitoring or cardiac rehabilitation programs that provide individualized postoperative advice or automated alerts during the 30-day follow-up. Use of generic wellness applications such as step counters or diet trackers will not be restricted, but any concurrent digital interventions with potential impact on readmission risk will be documented.

### Digital Access, Language Support, and Digital Literacy

Because Smart CABGuard relies on a smartphone app and a remote ECG device, access to a smartphone with reliable internet connectivity is an explicit eligibility criterion and practical prerequisite for participation. At enrollment, the research nurse will confirm device availability and network coverage, assist with app installation and ECG pairing, and complete a brief test check-in. The chatbot will support interaction in 1 or more locally relevant languages, with participants choosing their preferred language; where reading ability is limited, caregivers may assist with text input, and nurses can provide additional phone-based guidance. Basic digital literacy (ability to unlock the phone, open the app, and respond to prompts with or without caregiver support) will be assessed using a simple practical demonstration, and structured training with clear verbal instructions and pictorial guides will be provided to reduce barriers to engagement. The protocol acknowledges that these requirements may exclude some patients with very limited digital access or literacy, and this potential selection bias is addressed in the limitations.

### AI Intervention Framework

#### Algorithm Architecture and Version Governance

The system uses a Clinical Prediction with Large Language Models framework, in which structured and unstructured patient data are converted into standardized natural-language inputs for prediction tasks. A pretrained 7B-parameter model (Mistral-7B-v0.1) is adapted using parameter-efficient fine-tuning (low-rank adaptation). A fixed few-shot prompting strategy defines the patient context, the prediction objective (30-day readmission), and structured outputs (a risk score with an explanation). To ensure deterministic behavior, decoding parameters are fixed (temperature 0.2, top-p 0.9, and maximum tokens 512). Inputs are provided in structured templates, and outputs are constrained to predefined formats. The LLM is used solely for clinical decision support, and all outputs are checked by qualified clinicians before use.

The AI framework pairs a frozen, medically tuned LLM with a deterministic CRI derived from logistic regression. The LLM will remain unchanged throughout the trial to avoid model drift. All prompts, coefficients, and decision thresholds will be version-controlled and finalized before trial initiation. Any modification will require ethical approval and formal documentation.

#### Clinical Use Case for Smart CABGuard

Smart CABGuard is intended as a nurse-led decision support without autonomous treatment changes, and all outputs are reviewed by clinicians. It is designed to provide continuous symptom surveillance, rhythm monitoring, and risk-stratified recommendations by a trained cardiac nurse during the first 30 days after CABG surgery, with the explicit aim of reducing unplanned hospital readmissions.

#### Implementation Requirements Across Settings

On-site, Smart CABGuard requires secure institutional network access, EHR connectivity, and nurse workstations or tablets with the triage dashboard. Off-site, participants need an Android smartphone with internet, the Smart CABGuard app, and a Bluetooth-enabled single-lead ECG device. All components connect via a secure Trusted Research Environment that manages data exchange between the ECG device, app, AI engine, risk model, and nurse dashboard.

#### Human-AI Interaction and User Training

Smart CABGuard is designed as a nurse-led decision support tool. Only registered nurses with at least 1 year of experience in cardiac or cardiothoracic surgery units, and attending physicians in the Department of Cardiovascular and Thoracic Surgery, are authorized to review AI outputs and act on triage recommendations. All authorized users will complete a structured training module (approximately 2 hours) covering interpretation of CRI scores and risk categories, review of LLM-generated explanations, ECG alert management, and escalation algorithms. The research nurse and research assistants responsible for day-to-day triage and escalation in the Smart CABGuard arm will complete this training module and pass competency assessments before independently handling alerts. The training will include case-based simulations and competency checks to ensure that nurses and physicians understand the system’s capabilities and limitations, including the requirement to override or disregard AI recommendations when they conflict with clinical judgment.

#### Data Inputs and Model Construction

Structured EHR variables and deidentified clinical summaries will be processed at discharge within a secure Trusted Research Environment. High-impact predictors (eg, left ventricular ejection fraction and prior stroke) will not undergo imputation. If critical data are missing, the system will issue an “incomplete data advisory” and downgrade prediction confidence, prompting clinician review. The model output includes CRI score, risk category (low, moderate, and high), recommended triage intensity, and clinician-oriented natural-language explanation. The system functions strictly as CDS. Final treatment decisions remain under the clinician’s authority.

#### Input Data Quality Checks and Missing Data Handling

The AI intervention applies explicit input data eligibility criteria and continuous quality checks in addition to participant-level inclusion and exclusion. During follow-up, the chatbot delivers daily structured symptom check-ins, and the wearable ECG device records single-lead rhythm strips at scheduled times and on demand. Only perioperative EHR records containing predefined core variables and passing basic consistency checks are used for CRI-based predictions; the full list of input data variables extracted from the EHR is provided in Multimedia Appendix 3. Incomplete or inconsistent records are excluded, flagged for clinician review, and generate an incomplete data advisory with downgraded prediction confidence.

During follow-up, only fully completed check-ins reporting symptoms, medication adherence, and wound status in supported languages are considered eligible for analysis. ECG segments that meet predefined signal-quality thresholds are processed by the LLM and ECG pipeline, while poor-quality or unsupported inputs trigger repeat recording or charging prompts. If daily check-ins are missed for more than 48 hours, automated reminders are sent, followed by nurse phone contact after 72 hours of persistent nonadherence, with reasons documented. AI-generated risk estimates and triage recommendations are used only when these input data eligibility and quality criteria are satisfied.

#### Continuous Remote ECG Monitoring

The intervention incorporates a single-lead wearable ECG device (Amrita Spandanam, Amrita Vishwa Vidyapeetham, India), which records clinical-grade ECG via skin contact electrodes and streams data via Bluetooth to the patient’s Android smartphone for secure transmission to the Smart CABGuard nurse dashboard. The device and remote-monitoring framework, developed within the Amrita-Spandanam/AIM-Vitals platform, are compliant with ANSIAAMI EC13-2002 and IEC 60601-2-25/60601-2-27 standards for diagnostic ECG systems and are protected under US Patent 10,542,889 (Systems and Methods for Remote Health Monitoring and Management). Prior studies have shown satisfactory ECG signal quality and clinical usability across in-hospital treadmill testing, ambulatory monitoring, and remote rural deployments, with performance comparable with standard clinical ECG devices. In an Internet of Things–based smart-edge remote-monitoring study using this platform, downstream alerting models achieved high diagnostic performance for cardiac conditions (precision 0.87, recall 0.83, and *F*_1_-score 0.85), and deep-learning models trained on Amrita Spandanam ECG data for arrhythmia detection have demonstrated acceptable multiclass classification performance (*F*_1_-scores around 0.71 on benchmark datasets), supporting its suitability for continuous, safety-focused rhythm surveillance in resource-limited settings [49-51].

#### ECG Device Operation and Patient Setup

The wearable device can be worn on the wrist or carried in a pocket and connects via Bluetooth to a mobile app on the patient’s phone, with location services enabled for continuous monitoring. It should be removed only during bathing. Each patient will receive 2 devices: 1 active unit with a 24-hour battery life and 1 spare, as charging each device takes approximately 5‐6 hours. The troubleshooting steps for the Amrita Spandanam ECG device are provided in Multimedia Appendix 4. In accordance with the signed informed consent, all enrolled participants will return the remote ECG-monitoring devices at the first preplanned follow-up visit within 30 days after surgery.

#### AI Outputs and Clinical Use

For each enrolled participant, the AI system produces (1) a numerical CRI score, (2) a categorical risk label (low, moderate, or high), (3) a natural-language explanation summarizing key drivers of risk, (4) triage recommendations specifying suggested monitoring intensity and actions, and (5) ECG-based alert flags for arrhythmias of interest. These outputs are displayed to the nursing team on the triage dashboard and are used to prioritize follow-up calls, schedule expedited clinic reviews, and prompt urgent physician assessment or emergency referral when indicated. Final decisions regarding diagnostic workup, hospital readmission, and treatment modifications remain the responsibility of the treating clinicians, who may override or disregard AI recommendations whenever they conflict with clinical judgment.

### Outcomes

#### Outcomes Overview

The primary outcome is the 30-day all-cause unplanned hospital readmission rate following the index CABG admission. The 30 days will be measured from the date of surgery. Readmission is defined as any unplanned inpatient admission within 30 days of surgery; planned admissions are excluded. Emergency department or outpatient visits without admission will not be considered readmissions but will be captured as secondary outcomes. Secondary outcomes include time to detection of postoperative complications and postoperative morbidity, assessed using the validated 13-domain Cardiac Postoperative Morbidity Score (C-POMS) [52]. Given its limited psychometric validation in Indian post-CABG cohorts, it will be used as a secondary outcome, and its association with clinical end points (eg, length of stay and readmission) will be explored.

#### Outcome Ascertainment and Readmission Adjudication

All potential 30-day readmissions and emergency visits will be identified from institutional EHRs; structured follow-up telephone calls on postoperative days 7, 14, 21, and 30; and patient or caregiver self-reports. Two clinicians (a cardiac nurse and a cardiothoracic surgeon or cardiologist), blinded to trial allocation, will independently review discharge summaries, admission records, and other source documents using a predefined adjudication form to determine whether events meet the protocol definition of 30-day all-cause unplanned readmission. Discrepancies will be resolved by consensus, with a third adjudicator consulted if required. For patients admitted to nonindex hospitals, records will be requested with participant consent. If documentation cannot be obtained, events will be classified using the best available information and flagged as “probable” readmissions for sensitivity analyses. Events will be categorized as “readmission,” “planned admission,” “ED or outpatient visit without admission,” or “no event,” without further formal cause-of-readmission adjudication beyond descriptive reporting.

### Statistical Analysis

All analyses will be conducted using SPSS (version 30; IBM Corp). For the primary outcome (30-day all-cause unplanned readmission), statistical significance will be set at *P*<.10 (2-sided), consistent with the exploratory design and the sample size calculation. For all secondary outcomes, α=.05 (2-sided) is applied. For key predictors contributing to the CRI, missing values will not be imputed; records with missing data in these variables will be excluded from the CRI model development and validation analyses.

#### Phase I

The CRI will be derived from a multivariable logistic regression model, restricted to a maximum of 7‐8 predictors based on the ≥10 events per variable criterion (≈75 available outcome events). Internal validation will use 1000 iteration bootstrap resampling; temporal validation will use the 2023‐2024 holdout cohort. Model performance will be reported using ROC-AUC, PR-AUC, calibration plots, Hosmer-Lemeshow test, and Brier score. Clinical usefulness will be assessed by decision curve analysis. The risk classification threshold (low/moderate/high) will be selected based on net benefit across a probability range of 0.05‐0.30 and frozen before phase II. The LLM-based model will be formally compared with the logistic regression CRI using DeLong’s AUC comparison, net reclassification improvement, and integrated discrimination improvement on the same temporal validation set. Shapley Additive Explanations values will identify key predictors. Interrater agreement for PEMAT-A/V reviews will be assessed using the intraclass correlation coefficient.

#### Phase II

The BUS total and subscale scores will be reported as means and SDs or medians and IQRs, depending on the normality of data distribution. The proportion meeting the prespecified usability threshold (≥80%) will be reported. CSQ-8 total scores will be summarized with mean and SD; the proportion scoring ≥26 or in intermediate or high satisfaction categories will be reported against a target of ≥75%. Associations between usability or satisfaction scores and engagement measures will be examined using appropriate parametric or nonparametric tests.

#### Phase III

All randomized participants will be analyzed according to the intention-to-treat principle. Baseline comparability will be assessed using chi-square or Fisher exact test and 2-tailed *t* test or Mann-Whitney *U* test.

- Primary outcome: Thirty-day readmission proportions will be compared using chi-square or Fisher exact test. The primary effect will be reported as risk difference and risk ratio with 95% CI. Adjusted analysis will use multivariable logistic regression with age, sex, left ventricular ejection fraction, diabetes mellitus, number of bypass grafts, and CRI risk category at discharge.
- Secondary outcomes:
  1. C-POMS (7,14,21,30): linear mixed-effects model with group × time interaction; adjusted mean scores compared at day 30.
  2. Complication rates: chi-square or Fisher exact test with 95% CI.
  3. Time to complication recognition: independent samples 2-tailed *t* test or Mann-Whitney *U* test with 95% CI.
  4. Usability or Satisfaction (BUS, CSQ-8): descriptive summary for intervention arm; proportion meeting ≥80% BUS and ≥26 CSQ-8 targets reported.
- Missing data: If >5% of primary outcome data are missing, 2 sensitivity analyses will be performed: best-case analysis (all missing participants classified as not readmitted) and worst-case analysis (all missing participants classified as readmitted).

Process indicators, such as alert frequency, response time, and chatbot engagement, will be explored as potential mediators of outcomes. For the randomized controlled trial, all randomized participants with available 30-day readmission data will be included in the primary analysis. Missing primary outcome data will be minimized through structured follow-up and EHR verification.

### Ethical Governance and Regulatory Oversight

A first interim safety review will be performed after the first 15 participants (following the 30-day follow-up) to monitor for serious adverse events, unexpected device-related complications, and AI-related triage failures. If this review identifies any serious, unanticipated, and probably or definitely intervention-related harm (eg, death or life-threatening clinical deterioration attributable to delayed or inappropriate AI-guided triage in ≥2 participants), the data safety monitoring board (DSMB) will recommend temporary suspension of the intervention arm and initiate an urgent protocol review. If no such safety signal is identified, the trial will proceed as planned to subsequent interim analyses.

### Ethical Considerations

The study has received approval from the Institutional Ethics Committee–approval number EC/NEW/INST/2023/KL/0379–of the participating tertiary care hospital. All participants will sign a written informed consent form before joining the study. The consent form will clearly explain that the chatbot is an experimental AI tool for helping with clinical decisions, describe what continuous remote ECG monitoring involves, and explain how data will be collected, stored, and possibly used for other purposes. It will also explain possible risks, including worries about data privacy and the limits of digital devices. Participants will be told that the AI system helps with decisions but does not replace the judgment of their health care providers. If major changes to the study affect what participants experience or their level of risk, the consent form will be updated and approved again. Participants can leave the study at any time, and this will not affect the usual care they receive. The process of withdrawal, any changes from the planned protocol, and the reasons for stopping will be recorded in the case report forms. Data collected before a participant withdraws may still be used in deidentified form for analysis, unless the participant clearly asks for their data to be removed, and this is allowed by local regulations. Study data will be stored and handled in a secure research environment that uses strong encryption to keep information confidential and protected. Access to the AI chatbot intervention will stay under the control of the institution for the entire study period. The source code will not be publicly shared during the trial, but it can be reviewed by ethics committees and regulatory authorities if they request an audit.

### Ancillary and Posttrial Care

In line with the Declaration of Helsinki and national ethical guidelines, any harm directly attributable to trial procedures will be managed with appropriate medical care and compensation for research-related injury, as per institutional policy. There are no specific plans to provide ancillary or posttrial care beyond standard institutional follow-up, because the trial does not involve withdrawal of effective therapy or exposure to additional clinical risks beyond usual postoperative management.

### AI Governance and Accountability

The LLM component will remain in a frozen, non–self-learning configuration throughout the trial to prevent model drift and ensure reproducibility. All prompts, decision thresholds, and CRI coefficients will be version-controlled and formally documented. Any algorithmic modification after this amendment will require technical validation, documented version updates, independent review, and prior ethics committee approval before deployment. Clinical responsibility will rest entirely with the treating physicians and cardiac nursing team, as the AI system will not independently start treatment, change prescriptions, or overrule clinical decisions.

### Safety Monitoring and Adverse Event Reporting

A predefined safety-monitoring plan will describe the procedures for identifying and reporting adverse events, including device-related technical problems, delayed alert transmission, and misclassification of AI-generated risk. An independent DSMB conducts periodic reviews of the accumulating data. The DSMB will review unblinded safety reports at 25%, 50%, and 75% of the planned participant enrollment, with additional ad hoc meetings convened as necessary to evaluate adverse events and potential AI-related system failures.

An AI performance error is operationally defined as a clinically important mismatch between the AI system’s output and the observed clinical course, including (1) failure to generate a high-risk alert or escalation recommendation within 48 hours before a serious postoperative complication or unplanned readmission (potential false-negative triage), and (2) repeated high-risk alerts in the absence of any supporting clinical deterioration, device malfunction, or new diagnosis (potentially burdensome false-positive triage). Potential AI performance errors will be summarized in standardized incident forms and reviewed by the DSMB at scheduled meetings to determine whether they reflect systematic model limitations, data quality issues, or user interaction problems. Serious adverse events will be reported to the ethics committee within the mandated timelines. Any safety signal attributable to the AI system or the ECG-monitoring platform will trigger an immediate review and, if necessary, a temporary suspension of the intervention arm pending investigation.

### Transparency and Posttrial Data Sharing

After the trial is completed, deidentified data and the key documents describing the algorithm may be shared with qualified researchers under data use agreements, as follows by institutional policies, ethics, and privacy rules. Any secondary analysis of these data will need prior review and approval from the relevant ethics committee.

## Results

Ethics approval for this study was obtained from the Institutional Ethics Committee of Amrita Institute of Medical Sciences, Kochi, in April 2025, and the trial was registered with the Clinical Trials Registry of India in June 2025. Phase I retrospective data extraction commenced in May 2025 and is projected to be completed by April 2026, with the post-CABG patient needs survey running from June 2025 to January 2026. Phase II usability evaluation is projected for May to July 2026, followed by phase III participant recruitment from August 2026 to September 2027, and the main trial results are expected to be submitted for publication in early 2028.

The retrospective phase has been completed using EHR data. Analysis of the retrospective dataset using multivariable logistic regression has been completed, and development of the LLM-based component is currently in progress. Overall system development is at an intermediate stage. The study has received approval from the Institutional Ethics Committee of the participating tertiary care hospital (approval number EC/NEW/INST/2023/KL/0379).

## Discussion

### Expected Impact of the Proposed Study

This protocol outlines a rigorously structured, multiphase evaluation of Smart CABGuard, a nurse-led, LLM-based CDS chatbot integrated with continuous ECG monitoring for patients undergoing CABG. The planned RCT is designed to determine whether AI-assisted postoperative surveillance reduces 30-day hospital readmission compared with standard discharge care and to explore its effects on postoperative morbidity, patient-reported satisfaction, and health service usage.

The anticipated contribution of this study is to generate high-quality prospective evidence on the clinical usefulness, safety, and implementation feasibility of an LLM-enabled, nurse-led postoperative monitoring strategy within a high-volume tertiary cardiac center in an LMIC.

### Comparison With Prior Work

A recent scoping review [53] examined mobile, noninvasive strategies for detecting atrial fibrillation after discharge following cardiac surgery and found that wearable and handheld ECG devices can achieve high diagnostic accuracy, enable earlier AF detection, and reduce readmissions, although long-term effectiveness and optimal implementation still require further study. Many of these interventions also appeared to improve patient engagement.

Digital health interventions for postoperative cardiac care have mainly focused on telemonitoring, mobile health apps, and structured telephone follow-up. Several remote-monitoring studies have shown that detecting arrhythmias and early complications after cardiac surgery is feasible, but most do not incorporate integrated predictive models or conversational AI components [54-56].

Traditional readmission prediction tools in cardiac surgery have largely used logistic regression-based risk scores derived from structured clinical variables. Although these models can achieve moderate discrimination, they are typically static, difficult to apply at the time of discharge, and limited in their ability to incorporate unstructured contextual information. More recent AI-based readmission models [57] have improved performance but are often retrospective and have not been tested prospectively in randomized clinical trials.

The use of LLM-based conversational interfaces in postoperative care is still emerging. Most chatbot apps in health care focus on patient education, symptom screening, or mental health support, and few combine predictive risk stratification, physiologic telemetry integration, and nurse-led escalation within a single system. Prospective randomized evidence on LLM-enhanced CDS in surgical populations remains scarce.

This protocol builds on earlier work by integrating predictive modeling directly into routine patient-clinician interactions and evaluating its clinical impact in an RCT. The system uses a frozen LLM configuration with predefined prompts and version control to reduce the risk of model drift and to support reproducibility. By incorporating explainability and keeping final decisions under clinician control, the study is consistent with current guidance on the responsible use of AI in health care.

### Limitations

Several limitations should be noted. First, the study takes place in a single high-volume tertiary care hospital, which may limit how well the findings apply to lower-resource or community settings where discharge practices, digital literacy, and remote ECG capacity can differ. Second, as the trial is open-label, which is common in digital health interventions, there is a possibility of performance bias. Although outcome assessors and statisticians are blinded, participants and clinical staff are aware of allocation. Behavioral changes due to more frequent contact in the intervention arm may partly explain the effects we observe.

Third, the need for smartphone and internet connection may lead to selection bias by excluding people with limited digital access or literacy. Even with structured onboarding and nurse support, some differences in how participants engage with the digital tools are likely to persist. Fourth, although the LLM component is kept fixed to prevent model drift, LLMs can still show variability in language output and may require careful monitoring during deployment. Despite standardized training and prespecified escalation algorithms for the research nurse and research assistants, some variability in triage decisions and response times is anticipated and will be captured through process measures such as alert response intervals and adherence to escalation pathways. We address this by using deterministic risk scores, set prompt templates, and human oversight, but small differences in the chatbot’s phrase responses may still occur.

Finally, the use of α=.10 for the primary outcome reflects the exploratory nature of this first-in-class trial of an LLM-based intervention. While this reduces the risk of a false-negative finding, it increases the probability of a false-positive result compared with the conventional confirmatory threshold of α=.05. Results should therefore be regarded as hypothesis-generating, and a future adequately powered confirmatory trial with α=.05 will be required before clinical recommendations can be made.

### Conclusions

This protocol describes a step-by-step evaluation of an LLM-based clinical decision support chatbot that is linked with continuous ECG monitoring for patients after CABG surgery. It brings together risk prediction, transparent and accountable use of AI, nurse-led triage, and a randomized trial to provide strong data on how useful AI-assisted postoperative monitoring can be in real clinical practice.

If Smart CABGuard proves to be effective, it could offer a scalable way to lower readmissions, detect complications earlier, and increase patient participation in their recovery after cardiac surgery. In addition to CABG, the overall study design, combined risk modeling, use of conversational AI, connection with physiological monitoring, and strong ethical safeguards could act as a model for using generative AI safely in other high-risk surgical and medical settings. The results of this trial will add to the growing body of evidence on real-world use of LLM-based digital health tools and help guide future regulatory, clinical, and implementation standards for AI-supported postoperative care.

## Supplementary material

10.2196/103717 Multimedia Appendix 1 Integrated post–coronary artery bypass grafting triage plan.

10.2196/103717 Multimedia Appendix 2 Sample size sensitivity analyses.

10.2196/103717 Multimedia Appendix 3 Input data variables extracted from electronic health records for Complication Risk Index (CRI) model development and CRI-based predictions in the Smart CABGuard system.

10.2196/103717 Multimedia Appendix 4 Troubleshooting procedure for the Amrita Spandanam wearable electrocardiographic device used in the Smart CABGuard remote monitoring system.

10.2196/103717 Checklist 1 SPIRIT-AI checklist.

10.2196/103717 Peer Review Report 1 Grant peer review report.

## Abbreviations

BUS: chatBot Usability Scale
C-POMS: Cardiac Postoperative Morbidity Score
CABG: coronary artery bypass grafting
CDS: clinical decision support
CONSORT: Consolidated Standards of Reporting Trials
CRI: Complication Risk Index
CSQ-8: Client Satisfaction Questionnaire (8-item version)
DSMB: data safety monitoring board
ECG: electrocardiographic
EHR: electronic health record
LLM: large language model
LMIC: low- and middle-income country
mERA: mHealth Evidence Reporting and Assessment
PEMAT-A/V: Patient Education Materials Assessment Tool for Audiovisual Materials
PR-AUC: precision-recall area under the curve
RCT: randomized controlled trial
ROC-AUC: receiver operating characteristic area under the curve
TRIPOD: Transparent Reporting of a multivariable prediction model for Individual Prognosis Or Diagnosis
