# Supplementary material for: Nurse-Led Large Language Model Chatbot for Predicting and Preventing Complications After Coronary Artery Bypass Grafting: Protocol for a Randomized Controlled Trial
Source: JMIR Res Protoc. 2026 Aug 4;15:e103717. doi: 10.2196/103717 (PMC13436796; doi:10.2196/103717)
Supplement: Supplementary file 1 [file resprot-v15-e103717-s006.pdf]

Submission 21650

Reviewers' summary of the major strengths and the major limitations of the proposal.

Review 1:
This study is valuable to develop a a nurse-led AI chatbot for CABG patients. The methodology is well described. However, the sample size proposed in the Phase 3 RCT exceeds 700 participants, raising concerns about whether the researchers can successfully complete the study. Thank you.

Review 2:
Innovative work of great interest and well presented

Review 3:
The proposal is well-written, and the application of Neuman's theory has enhanced its quality. The methods used are appropriate and have been effectively integrated with the theoretical framework. The research presents an innovative and novel idea, which is commendable.
