# Supplementary material for: Nurse-Led Large Language Model Chatbot for Predicting and Preventing Complications After Coronary Artery Bypass Grafting: Protocol for a Randomized Controlled Trial
Source: JMIR Res Protoc. 2026 Aug 4;15:e103717. doi: 10.2196/103717 (PMC13436796; doi:10.2196/103717)
Supplement: Supplementary file 2 [file resprot-v15-e103717-s001.docx]

**Multimedia Appendix 1**

**Plan for Integrated Post-CABG Chatbot Triage with Remote ECG Monitoring**

*This appendix presents the structured three-zone (Green/Amber/Red) triage protocol used by the Smart CABGuard nurse-led chatbot system, integrating remote ECG monitoring across clinical, surgical, activity, monitoring, psychological, and special population domains for post-CABG patients during the 30-day post-discharge surveillance period.*

| **Domain / Care Component** | **GREEN Self‑care & Routine Review** | **AMBER Same‑Day Nurse Review** | **🔴 RED Immediate Emergency Care** |
| --- | --- | --- | --- |
| **TRIAGE SYMPTOMS** | | | |
| **Core symptoms** | Mild incisional or leg‑site pain; able to do daily activities; mild cough; sleep/psychological concerns only. | Breathless on walking; chest heaviness; markedly tired; increasing leg swelling without breathlessness; gas trouble with chest discomfort; poor oral intake; symptomatic sugar fluctuations but otherwise stable. | Breathless at rest or cannot lie flat; sudden severe chest pain; swelling with breathlessness; repeated vomiting with weakness; collapse or unresponsiveness. |
| **ECG – VT** | – | – | Any VT alert from remote ECG (extending up to ~1 minute) → classify as Red and advise urgent hospital visit. |
| **ECG – AF** | – | New AF persisting ≥15 minutes without chest pain, severe breathlessness, or syncope → nurse to review same day. | AF with severe breathlessness, chest pain, syncope, or hypotension symptoms → emergency evaluation. |
| **Pain score (0–10)** | 0–3: expected post‑surgical or leg‑site pain, stable or improving, no other warning symptoms. | 4–6: new or increasing chest/wound/leg pain disturbing sleep or activities but without red‑flag features. | 7–10: sudden severe chest or wound/leg pain, or pain with breathlessness, sweating, or feeling faint; excessive pain from surgical wound. |
| **Breathlessness level** | Only on fast walking or climbing stairs; settles with rest; no chest pain or wheeze. | On normal walking but comfortable at rest. | Breathless at rest or when lying flat; severe shortness of breath. |
| **Heart rate** | 50–100 beats/min, regular, no palpitations or concerning symptoms. | 41–49 or 101–120 beats/min with mild palpitations or mild dizziness but otherwise stable. | Heart rate very low or very high with severe dizziness, syncope, chest pain, or severe breathlessness. |
| **Blood pressure monitoring** | Regular home monitoring; within individual target range. | Persistently elevated (SBP >150 or DBP >95) or low (SBP <90) without severe symptoms → nurse review for medication adjustment. | Symptomatic hypotension (severe dizziness, confusion, syncope) or hypertensive emergency with chest pain/breathlessness. |
| **Blood sugar (if diabetic)** | 90–180 mg/dL, asymptomatic; FBS <120 mg/dL, PPBS <200 mg/dL. | 70–89 or 181–250 mg/dL with mild symptoms (tiredness, mild tremor, mild polyuria). Nurse adjusts counselling/medications. | Persistently >200 mg/dL with vomiting or altered sensorium; marked hypoglycaemia with confusion or inability to take orally. |
| **Temperature / fever** | Up to 99.0°F with only mild body pain or tiredness. | 99.1–100.0°F with cough, wound pain, burning micturition, or loose stools but clinically stable. | Fever >100°F with rigors, confusion, associated Red symptoms (severe breathlessness, hypotension), or signs of wound infection. |
| **Consciousness / mental status** | Fully alert, oriented, no fainting; anxiety or poor sleep only. | Near‑fainting episodes, new confusion, or extreme sluggishness but still responsive; giddiness without other red flags. | Loss of consciousness, collapse, unresponsiveness, or rapidly worsening confusion. |
| **SURGICAL SITE CARE** | | | |
| **Wound / chest incision** | Mild incisional discomfort improving over time; dry wound with no discharge. Apply T-bact ointment daily × 1 week; daily dressing with Betadine solution. | Mild redness, warmth, or minimal serous discharge from wound but no fever or systemic symptoms → same‑day wound check. | Any purulent discharge, excessive bleeding, wound dehiscence, spreading redness, or wound problems with fever/systemic signs. |
| **Leg swelling / vein‑harvest site** | Mild leg swelling or discomfort improving over days, no redness or warmth. | Increasing swelling, mild redness or warmth, difficulty walking but no severe pain and no breathlessness. | Sudden marked swelling with severe pain and redness, or leg swelling with breathlessness (suspected DVT/PE). |
| **Sternal support** | Chest binder worn as advised; no sternal instability or clicking. | Mild sternal discomfort or concern about binder fit → nurse to review and adjust. | Sternal instability, clicking, or separation with severe pain. |
| **ACTIVITY & LIFESTYLE** | | | |
| **Physical activity** | Gradual activity resumption; restricted heavy activities for 3 weeks; avoid pressure on arm/chest/shoulders. | Excessive fatigue limiting daily activities despite adherence to restrictions → nurse counselling and review. | Unable to perform basic self‑care activities; severe weakness. |
| **Respiratory care** | Regular spirometry exercises as instructed. | Poor compliance or difficulty with exercises but no breathlessness at rest → nurse education and support. | Respiratory distress or severe breathlessness despite spirometry. |
| **Diet** | Low‑fat, high‑protein diet; adequate hydration. | Poor oral intake, nausea, or difficulty eating but tolerating fluids. | Persistent vomiting, unable to tolerate oral intake, or vomiting with weakness. |
| **MONITORING & MEDICATIONS** | | | |
| **Anticoagulation (if applicable)** | INR monitoring as scheduled; INR within therapeutic range (typically 2.0–3.0). | INR subtherapeutic or mildly supratherapeutic without bleeding → nurse to adjust warfarin dose. | Bleeding from any site (especially if on anticoagulants); INR critically high with bleeding risk. |
| **Medication adherence** | Taking all prescribed medications regularly. | Questions about medications, side effects, or missed doses → nurse review and counselling. | Adverse drug reaction with severe symptoms (e.g., severe rash, angioedema, hypotension). |
| **PSYCHOLOGICAL & SLEEP** | | | |
| **Sleep / psychological status** | Early post‑operative anxiety, poor sleep, or need for emotional support without physical red flags. | Persistent insomnia with worsening fatigue and poor functioning but no major physical symptoms → nurse counselling or referral. | Severe agitation, confusion, suicidal ideas, or insomnia with inability to function. |
| **SPECIAL POPULATIONS** | | | |
| **Dialysis patients** | Continue hemodialysis as scheduled in local hospital; AVF care as instructed. | Difficulty accessing dialysis or concerns about vascular access → nurse coordination. | Missed dialysis with severe fluid overload, breathlessness, or altered mental status. |
| **Physiotherapy needs** | Daily physiotherapy as per physiotherapist's advice (if prescribed for specific problems like shoulder stiffness). | Poor progress or difficulty with physiotherapy exercises → nurse review and physiotherapy referral. | Severe pain or limitation preventing any movement with associated symptoms. |

**Emergency Contact Instructions**

In the event of any Red‑zone symptom (fever >100°F, vomiting, bleeding, shortness of breath, chest pain, or worsening symptoms), patients must:
 • NOT drive themselves to the hospital
 • Contact emergency services immediately
 • Report to the nearest emergency department/casualty

**Notes for Chatbot Implementation**

1. The chatbot delivers structured prompts for each chief complaint (pain scale 0–10, breathlessness level, heart rate, blood sugar, temperature, and consciousness level).
2. Triage rules are applied hierarchically: a single Red‑zone criterion overrides any Green or Amber response.
3. Green‑zone patients receive automated self‑care education linked to discharge advice, with no immediate nurse action required.
4. Amber‑zone patients trigger an automatic same‑day nurse callback for clinical assessment and counselling.
5. Red‑zone patients receive immediate automated alerts with clear emergency contact information and explicit instructions not to delay seeking care.

*Footnote: Triage thresholds for ECG parameters (AF, VT), blood pressure, blood glucose, anticoagulation targets, and pain scoring are consistent with current international clinical guidelines [ESC AF Guidelines, 2021; ACC/AHA VA Guidelines, 2018; ADA Standards of Care, 2024; ESC/ESH Hypertension Guidelines, 2023]. Wound care and device‑specific protocols are institution‑derived based on standard post-CABG care practices at Amrita Institute of Medical Sciences, Kochi, India.*
