# Supplementary material for: Nurse-Led Large Language Model Chatbot for Predicting and Preventing Complications After Coronary Artery Bypass Grafting: Protocol for a Randomized Controlled Trial
Source: JMIR Res Protoc. 2026 Aug 4;15:e103717. doi: 10.2196/103717 (PMC13436796; doi:10.2196/103717)
Supplement: Supplementary file 3 [file resprot-v15-e103717-s002.docx]

***Multimedia Appendix 2***

| **Relative Reduction** | **Intervention Rate** | **n per arm** | **n per arm (after 10% attrition)** | **Total Sample** |
| --- | --- | --- | --- | --- |
| 35% | 6.96% | 801 | 880 | 1760 |
| 50%  (Current Assumption) | 5.36% | 361 | 400 | 800 |
| 70% | 3.21% | 160 | 175 | 350 |

α = 0.10 (two-sided), power = 80%, 10% attrition adjustment applied. Baseline 30-day readmission rate: 10.71% (Institutional data, Amrita IMS, Kochi). Sample size calculated using nMaster. Effect size assumptions informed by Downing et al. 2023 and Mentias et al. 2023. Calculated in G* Power
