# Supplementary material for: Nurse-Led Large Language Model Chatbot for Predicting and Preventing Complications After Coronary Artery Bypass Grafting: Protocol for a Randomized Controlled Trial
Source: JMIR Res Protoc. 2026 Aug 4;15:e103717. doi: 10.2196/103717 (PMC13436796; doi:10.2196/103717)
Supplement: Supplementary file 4 [file resprot-v15-e103717-s003.docx]

**Multimedia Appendix 3**

**Electronic Health Record (EHR) Input Data Variables**

| **Variable Category** | **Variables Extracted from EHR** |
| --- | --- |
| **A. PATIENT IDENTIFICATION & DEMOGRAPHIC DETAILS** | |
| **Patient demographics** | Age (years); Gender (male/female); body mass index (BMI, kg/m²); diagnosis; type of surgery; urgency of surgery (elective/urgent/emergency); date of admission; date of surgery; date of discharge; total hospital stay (days) |
| **B. PRE-OPERATIVE CLINICAL DETAILS** | |
| **Cardiac risk scores & functional status** | Pre-operative left ventricular ejection fraction (LVEF, %); EuroSCORE II; NYHA functional class; pre-operative LMCA stenosis >50% |
| **Comorbidities** | Diabetes mellitus (DM); hypertension (HTN); peripheral vascular/occlusive disease (PVOD); chronic obstructive pulmonary disease (COPD); pulmonary arterial hypertension (PAH); prior stroke; renal dysfunction; coronary artery disease (CAD); renal calculi; hypothyroidism; diabetic retinopathy; chronic liver disease (CLD) |
| **Pre-operative haemodynamic support** | Pre-operative inotrope use; pre-operative intra-aortic balloon pump (IABP); pre-operative mechanical ventilation; pre-operative ICU stay |
| **Pre-operative medications** | Pre-operative second-line antibiotics; all prescribed pre-operative medications (as documented in the EHR medication record) |
| **Pre-operative laboratory values** | FBS; urea; creatinine; creatinine clearance; total cholesterol; triglycerides; HDL; LDL; total bilirubin; SGOT; SGPT; creatine kinase (CK); T4; HbA1c; TSH; Na⁺; K⁺; total WBC count (TC); Hb; platelets; ESR; PT/INR; APTT; BNP; CK-MB; Troponin I; CRP; procalcitonin; D-dimer; urine routine; folic acid; vitamin B12; LDH; ANA; biopsy results (if applicable); sputum culture; blood culture; urine culture; rheumatology tests; pus/wound swab culture |
| **Pre-operative radiology & imaging** | USG abdomen; CT (chest/abdomen); MRI; nerve conduction study (NCS); Doppler studies; coronary angiography (CAG); echocardiogram; endoscopy |
| **C. PERI-OPERATIVE & POST-OPERATIVE DETAILS** | |
| **Intra-operative details** | Date of surgery; incision time; closure time; total duration of surgery (hours); operative findings and notes |
| **Post-operative ICU vital signs (Day of surgery; POD 1, 2, 3)** | Maximum heart rate (HR, bpm); respiratory rate (RR, /min); temperature (°F); mean arterial pressure (MAP, mmHg); systolic BP (mmHg); diastolic BP (mmHg); SpO₂ (%) |
| **Post-operative ICU alert thresholds (First alert time in hours after surgery)** | BP systolic >150 mmHg; BP systolic <90 mmHg; MAP <60 mmHg; MAP >90 mmHg; HR <60 bpm; HR >100 bpm; new-onset arrhythmia events; RR <12/min; RR >30/min; temperature >99°F; SpO₂ <95% |
| **Post-operative clinical events** | Total ICU stay (days); inotrope use duration; re-exploration (yes/no); renal failure requiring dialysis; post-operative ventilatory support (hours); pulmonary artery catheterization details; reintubation details; IABP use; central line duration (days); bloodstream infection; second-line antibiotic use; treatment for arrhythmias; post-operative pacing; post-operative BiPAP; readmission from ward back to ICU; blood loss within 24 hours (mL); number of blood products administered |
| **Post-operative laboratory values** | POD 1: Na⁺, K⁺, total bilirubin, SGOT, SGPT, ALP, procalcitonin, CRP, creatinine clearance POD 1 & 2: urea, creatinine, WBC, Hb, platelets Discharge day: urea, creatinine |
| **Post-operative imaging** | Post-operative echocardiography; post-operative USG (if indicated) |
| **Discharge details** | Post-operative advice on discharge; discharge medications after CABG |
| **D. READMISSION DETAILS — PHASE I RETROSPECTIVE OUTCOME DATA (Used to define 30-day unplanned readmission as the primary outcome variable for CRI model development)** | |
| **Readmission identification** | Date of readmission; total readmission hospital stay (days); readmission diagnosis / presenting complaints |
| **Readmission course in hospital** | Clinical course during readmission (as documented in EHR discharge summary) |
| **Readmission laboratory values** | Na⁺; K⁺; Hb; TC (total WBC count); platelets; ESR; procalcitonin; D-dimer; CK-MB; CRP; Troponin T; BNP; PT/INR; APTT; urea; creatinine; urine analysis; total bilirubin; SGOT; SGPT; ALP; creatinine kinase; RBS; FBS |
| **Readmission culture reports** | Blood culture; urine culture; pus/wound swab culture; sputum culture |
| **Readmission radiology/imaging** | USG; echocardiography; CT; MRI; Doppler; endoscopy/ERCP |
| **Medication on discharge after readmission** | All medications prescribed at discharge following a readmission episode |

**Data Completeness Criteria:** *An EHR record is considered incomplete and excluded from CRI model development if any of the following critical variables are missing: age, BMI, EuroSCORE II, pre-operative LVEF, pre-operative PT/INR, duration of surgery, total ICU stay duration, post-operative renal dysfunction status, or post-operative vital signs (POD 1–3). Incomplete records are flagged and generate an 'incomplete-data' advisory within the Smart CABGuard system during Phase III deployment, accompanied by a downgraded prediction confidence and a prompt for clinician review.*

**Note on Study Phase Context:** *All variables listed above are extracted exclusively from retrospective EHR records during Phase I (January 2020 – December 2024) for the development and temporal validation of the Complication Risk Index (CRI). The readmission-related variables in Section D represent the primary outcome of the CRI model (30-day all-cause unplanned readmission). These variables are not collected prospectively during Phase II (system usability evaluation) or Phase III (randomized controlled trial), where the CRI is deployed as a frozen, pre-validated tool within the Smart CABGuard platform.*
