# Supplementary material for: Nurse-Led Large Language Model Chatbot for Predicting and Preventing Complications After Coronary Artery Bypass Grafting: Protocol for a Randomized Controlled Trial
Source: JMIR Res Protoc. 2026 Aug 4;15:e103717. doi: 10.2196/103717 (PMC13436796; doi:10.2196/103717)
Supplement: Supplementary file 5 [file resprot-v15-e103717-s004.docx]

**Multimedia Appendix 4**

**Troubleshooting Guide for the Amrita Spandanam Wearable ECG Device and Smart CABGuard Application**

*This appendix provides a structured troubleshooting reference for patients, caregivers, and research nurses when technical issues arise with the Amrita Spandanam single-lead wearable ECG device or the Smart CABGuard mobile application during the 30-day post-discharge monitoring period in Phase II and Phase III of this study. The research nurse is responsible for first-line troubleshooting support; patients and caregivers are provided a simplified version of this guide during onboarding.*

| **No.** | **Issue** | **Possible Cause** | **Recommended Action** |
| --- | --- | --- | --- |
| **DEVICE CONNECTIVITY** | | | |
| **1** | **App not syncing with the ECG device** | *Bluetooth is turned off on the patient's smartphone; location services are disabled.* | Instruct the patient or caregiver to:  1. Enable Bluetooth on the smartphone.  2. Turn on location services for the Smart CABGuard app.  3. Close and reopen the app and attempt re-pairing. If unresolved within 1 hour, the research nurse will contact the patient by phone and remotely guide troubleshooting. Document the episode in the monitoring log. |
| **2** | **ECG signal not transmitting to the nurse dashboard** | *Smartphone not connected to the internet; ECG device out of Bluetooth range (>10 metres from phone).* | Advise the patient to:  1. Confirm active mobile data or Wi-Fi connection.  2. Keep the smartphone within 1–2 metres of the ECG device during monitoring.  3. Restart the app if the connection icon shows offline status. Research nurse to verify dashboard receipt of last transmission and note the gap in the monitoring log. |
| **DEVICE POWER & HARDWARE** | | | |
| **3** | **The ECG band battery is low, or the device is not powering on** | *Failure to adhere to the recommended charging schedule; the charging cable is not properly connected.* | During onboarding training, emphasise that the device must be charged continuously for 5–6 hours to achieve full battery life (~24 hours of monitoring). Smart CABGuard chatbot is configured to send a daily 'Charge Battery' reminder to the patient. Each participant receives two devices (one active, one spare) to ensure uninterrupted monitoring during charging periods. If the device fails to power on after a full charge, the research nurse will arrange same-day replacement from the spare device pool. |
| **4** | **Device not recording ECG / poor signal quality** | *Incorrect electrode placement; dry skin reducing skin–electrode contact; excessive patient movement during recording.* | Instruct the patient to:  1. Wear the device snugly on the wrist or as directed (skin-contact electrodes).  2. Ensure skin is clean and dry before recording.  3. Remain still for at least 30 seconds during on-demand recordings. Poor-quality ECG segments are automatically flagged by the system; the chatbot will prompt a repeat recording. Nurse to review signal quality flags on the dashboard. |
| **APPLICATION & DAILY CHECK-IN ISSUES** | | | |
| **5** | **No daily check-in logs received** | *Patient missed or dismissed daily chatbot prompts (non-adherence); smartphone notifications turned off.* | Smart CABGuard sends automated reminders if check-ins are missed for >48 hours. The research nurse initiates phone contact after 72 hours of persistent non-adherence. Reasons for non-adherence are documented in the case report form. During onboarding, confirm that app notifications are enabled on the patient's phone. |
| **6** | **Patient is unable to understand or respond to chatbot prompts** | *Limited digital literacy; language preference not matched; caregiver not present to assist.* | Confirm preferred language during onboarding and configure chatbot accordingly. If reading ability is limited, caregiver assistance with text input is permitted. A research nurse can provide additional phone-based guidance. For persistent difficulty, document it as a digital literacy barrier in the participant log and escalate to the principal investigator if it affects data completeness. |
| **ESCALATION & SYSTEM FAILURES** | | | |
| **7** | **AI system not generating risk alerts or triage output** | *Missing or incomplete EHR input data, incomplete check-in submission, or system server issue.* | The system automatically issues an 'incomplete-data advisory' and downgrades prediction confidence when core input variables are missing. Research nurse to manually review the participant's symptom check-in and complete a nurse-led clinical assessment by phone. Major technical failures that cannot be resolved within 72 hours and that materially compromise monitoring will be reported as protocol deviations and may trigger the temporary suspension of the digital intervention for that participant. |
| **8** | **Patient reports app errors or device damage** | *Accidental damage, software crash, or hardware fault.* | Patient or caregiver to contact the research nurse immediately via the provided emergency helpline number. Research nurse to document the issue, arrange a replacement device or app reinstallation within 24 hours, and log the incident in the safety monitoring record. If monitoring is interrupted for >72 hours due to the fault, this is recorded as a protocol deviation. |

**Note:** *This troubleshooting guide is referenced in Section ECG Device Operation and Patient Setup of the main manuscript. All troubleshooting episodes are documented in the participant monitoring log and reviewed by the research nurse and principal investigator at scheduled intervals. Recurring technical issues that affect data quality or participant safety are reported to the Data Safety Monitoring Board (DSMB) at scheduled interim reviews.*
